# Data on saponins, xylan and cellulose yield obtained from quinoa stalks after pressurized hot water extraction

**DOI:** 10.1016/j.dib.2018.08.003

**Published:** 2018-08-08

**Authors:** Alicia Gil-Ramirez, Daniel Martin Salas-Veizaga, Carl Grey, Eva Nordberg Karlsson, Irene Rodriguez-Meizoso, Javier A. Linares-Pastén

**Affiliations:** a Centre for Analysis and Synthesis, Department of Chemistry, Lund University, P.O. Box 124, 22100 Lund, Sweden; b Division of Biotechnology, Department of Chemistry, Lund University, P.O. Box 124, 22100 Lund, Sweden; c Instituto de Investigaciones Fármaco Bioquímicas, Universidad Mayor de San Andrés, La Paz, Bolivia

## Abstract

The data we present below are linked to our research paper “Integrated process for sequential extraction of saponins, xylan and cellulose from quinoa stalks (*Chenopodium quinoa* Willd.)” (Gil-Ramírez et al., 2018) [1]. The objective is to provide supplementary information in order to facilitate the comprehension of the central composite experimental design (rotatable 2^2^) used in the integrated process of extractions. Two factors, temperature and time of extraction are considered in the design. The responses are the yield of saponin, xylan and cellulose. First, the desirable linear regression obtained by the observed vs. predicted yields plot for each variable response confirm the validation of the model (Fig. 1). Second, the data presented here through Standardized Pareto Charts (Fig. 2), provides information about the effect of the time and temperature, as well as their interactions, in the yield of saponins, xylan and cellulose obtained in an integrated sequential extraction.

**Specifications Table**

**Table:** 

Subject area	*Chemistry, Biology, Engineering*
More specific subject area	*Biorefinery, valorization of bioproducts*
Type of data	*Graphs*
How data was acquired	*Spectrophotometric, gravimetric and High Performance Anion Exchange Chromatography with Pulsed Amperiometric Detection (HPAEC – PAD) analysis.*
Data format	*Analyzed, linear regression, standardized Pareto charts*
Experimental factors	*Temperature and time in the saponin extraction*
Experimental features	*Pressurized hot water extraction (PHWE) of saponins, alkaline extraction of xylan and acid recovery of cellulose.*
Data source location	*Lund University, Department of Chemistry, Division of Biotechnology. Sweden*
Data accessibility	*All data are available in this document.*
Related research article	*Gil-Ramirez, A., Salas-Veizaga, D.M., Grey, C., Nordberg Karlsson, E., Rodriguez-Meizoso, I., Linares-Pastén, J.A.(2018). Integrated process for sequential extraction of saponins, xylan and cellulose from quinoa stalks (Chenopodium quinoa Willd.)* Industrial Crops and Products, 121: 54–65 [1].

**Value of the data**

• The linear trend obtained by the observed vs. predicted yields plot for each variable response support the validation of the models elaborated for the extraction of saponins, xylan and cellulose (Fig. 1).

**Fig. 1:**
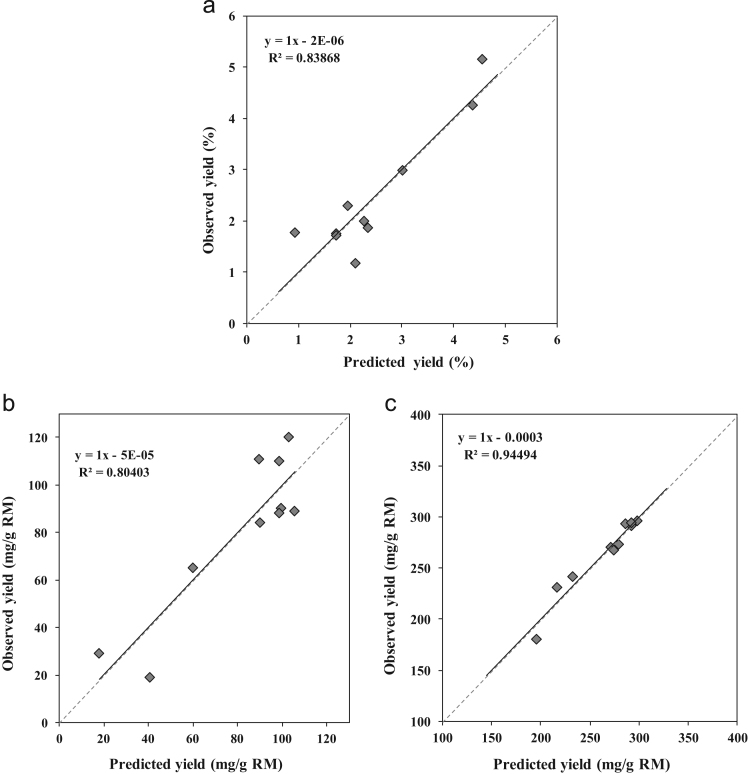
Regression plots generated using observed (in the *y*-axis) vs. predicted (in the *x*-axis) yields values of a) saponins, b) xylan and c) cellulose from quinoa stalks after PHWE. Dashed lines correspond to the perfect fit line (*y*-intercept = 0, slope = 1).
• Standardized Pareto Chart shows the effect of time and temperature of PHWE, as well as their interactions in the yield of saponins, xylan and cellulose obtained (Fig. 2).

**Fig. 2:**
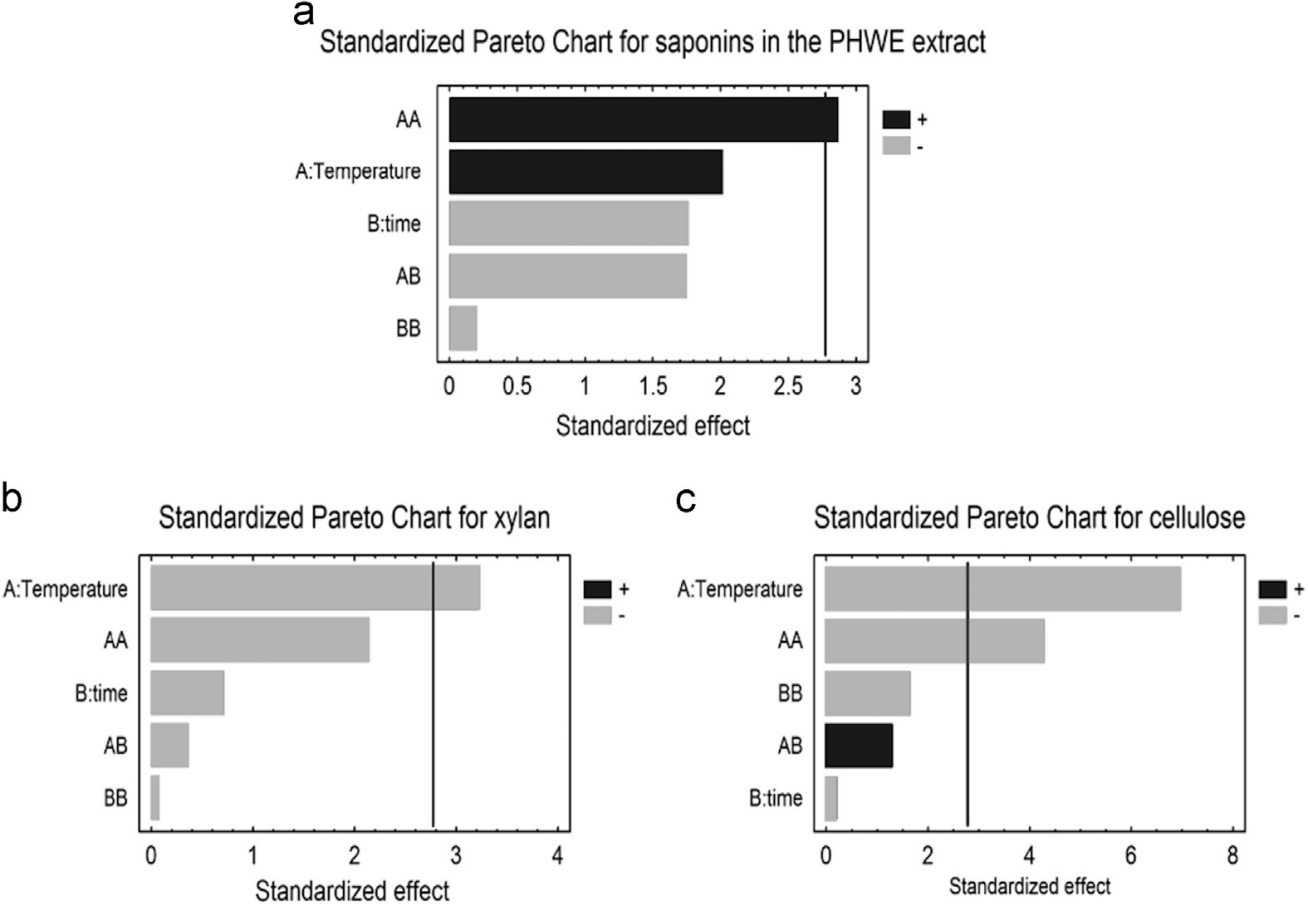
Standardized Pareto Chart for a) saponins, b) xylan and c) cellulose yield obtained from quinoa stalks after PHWE. The effects are sorted from most to least significance and the length of each bar is proportional to the standardized effect. The vertical line shows the location of the 0.05 critical value for Student׳s *t*-test. Any bar crossing the vertical line is statistically significant at a 95% of confidence level. Dark and light bars show positive and negative effects respectively.

## 1 Data

Linear regression obtained by the observed vs. predicted yields plot for each variable response allows the validation of the models obtained for the extraction of saponins, xylan and cellulose (Fig. 1). Pareto Chart shows the effect of factors, such as time and temperature of extraction, as well as their interactions (Fig. 2).

## 2 Experimental design, materials, and methods

The experimental design was a central composite and rotatable design 2^2^ with star points (*α* = 1.414) with two central points selected for the PHWEs of saponins. The factors studied were temperature (in a range of 50 to 170 °C) and time (in a range of 11–60 min) of extraction. Xylan was extracted by alkaline extraction with NaOH. Subsequently, cellulose was recovered by reflux in a mixture of acetic and nitric acid. Saponins were analyzed spectrophootometrically and by mass spectrometry. Xylan and eellulose were analyzed by High Performance Anion Exchange Chromatography with Pulsed Amperiometric Detection (HPAEC – PAD), Fourier transform infrared (FT-IR) spectroscopy and Scanning electron microscopy (SEM) [1].
