# Supplementary material for: Data on saponins, xylan and cellulose yield obtained from quinoa stalks after pressurized hot water extraction
Source: Data Brief. 2018 Aug 8;20:289–92. doi: 10.1016/j.dib.2018.08.003 (PMC6106705; doi:10.1016/j.dib.2018.08.003)
Supplement: Supplementary file 1 — Transparency document [file mmc1.docx]

**Transparency document**

**Data Article:** Data on saponins, xylan and cellulose yield obtained from quinoa stalks after pressurized hot water extraction

**Authors:** Alicia Gil-Ramirez, Daniel Martin Salas-Veizaga, Carl Grey, Eva Nordberg Karlsson, Irene Rodriguez-Meizoso, Javier A. Linares-Pastén

**Conflict of interest:** The authors declare that they have no conflict of interest.

**Ethical approval:** This data article does not contain any studies with human participants or animals performed by any of the authors.
